# Relative Importance of Social Status and Physiological Need in Determining Leadership in a Social Forager

**DOI:** 10.1371/journal.pone.0064778

**Published:** 2013-05-15

**Authors:** Markus Öst, Kim Jaatinen

**Affiliations:** 1 Aronia Coastal Zone Research Team, Åbo Akademi University and Novia University of Applied Sciences, Ekenäs, Finland; 2 Evolution, Ecology and Genetics, Research School of Biology, The Australian National University, Canberra, ACT, Australia; CNRS, Université de Bourgogne, France

## Abstract

Group decisions on the timing of mutually exclusive activities pose a dilemma: monopolized decision-making by a single leader compromises the optimal timing of activities by the others, while independent decision-making by all group members undermines group coherence. Theory suggests that initiation of foraging should be determined by physiological demand in social foragers, thereby resolving the dilemma of group coordination. However, empirical support is scant, perhaps because intrinsic qualities predisposing individuals to leadership (social status, experience or personality), or their interactions with satiation level, have seldom been simultaneously considered. Here, we examine which females initiated foraging in eider (*Somateria mollissima*) brood-rearing coalitions, characterized by female dominance hierarchies and potentially large individual differences in energy requirements due to strenuous breeding effort. Several physiological and social factors, except for female breeding experience and boldness towards predators, explained foraging initiation. Initiators spent a larger proportion of time submerged during foraging bouts, had poorer body condition and smaller structural size, but they were also aggressive and occupied central positions. Initiation probability also declined with female group size as expected given random assignment of initiators. However, the relative importance of physiological predictors of leadership propensity (active foraging time, body condition, structural size) exceeded those of social predictors (aggressiveness, spatial position) by an order of magnitude. These results confirm recent theoretical work suggesting that ‘leading according to need’ is an evolutionary viable strategy regardless of group heterogeneity or underlying dominance structure.

## Introduction

Animals in groups often must choose between mutually exclusive actions, which poses a dilemma because individuals differ in their preferences as to when and what to do next [1]. If all individuals follow their own preferences, group coherence is undermined, resulting in an unfavourable outcome for everyone. Conversely, entrusting leadership to a single individual in the group conflicts with the followers’ interests, because they are less likely to satisfy their preferences [2]. Understanding the emergence of leadership, defined as the ability to initiate group activities or movements [3], is therefore a key challenge in social biology [4].

Explanations for the emergence of leadership fall into two broad categories. First, individuals may possess unique intrinsic qualities such as social status, knowledge or a specific personality predisposing them to leadership [5]. Consistent leadership by specific individuals is most common in stable groups with a dominance hierarchy [6]. Alternatively, the individuals standing to gain the most if the group adopts their preferences may emerge as leaders. Such ‘leadership according to need’ (*sensu* [7]) is frequently based on physiological motivation to initiate activities. For example, Rands et al. [8], [9] showed theoretically that animals gain from synchronization of their feeding behaviour and the pace-maker should be the individual with the greatest physiological demands. The motivation of the well-fed individual to follow the initiator is based on predation risk reduction, whereas the behaviour of the individual with low reserves should be dictated solely by its own energy reserve. Consequently, synchronization of foraging activity and differences in the energetic reserves between the individuals spontaneously develop and may be moderately stable over time. While these models were originally developed for pairs of foragers, subsequent work has indicated that physiological need may determine leadership also in groups larger than two [7], [10].

Leadership has traditionally been portrayed as driven by intrinsic individual attributes, or else by differences in physiological state (but see [5], [11], [12]). However, in reality, collective decisions may be reached by a combination of different, not mutually exclusive rules [6]. The interactive nature of these effects [12] may explain why only meagre empirical evidence exists for the importance of energetic reserves as a predictor of leadership in social foragers [9], and, likewise, why consistent leadership where a single individual initiates every collective movement is rare in nature [13]. Confounding between the effects of individual asymmetries, physiological state and social interactions on leadership propensity may occur at several levels. First, the effects of social status and food deprivation on leadership propensity may be confounded. Thus, the dominant individual may be the sole breeder, and so its energy-consuming reproductive activity may also make it the hungriest individual [10]. Correspondingly, individual differences in satiation may override the effects of personality differences on the probability of taking on a leadership role [12]. Second, intrinsic asymmetries may be modulated by the social environment. For example, in larger groups, the effect of dominance [14] or boldness [15] on leadership propensity may be diminished, perhaps reflecting more equally shared decision-making in such groups (cf. [1]). However, generalizations are difficult because some individuals may gain a disproportionate weight in decision outcomes even in large groups where individuals cannot directly address all other group members [7], [16].

Future work will need to integrate the synergistic effects of multiple factors predicting leadership [12]. Following this call, we examined which individuals initiated foraging bouts in brood-rearing coalitions of female eider ducks (*Somateria mollissima*). This is an ideal study system due to the simultaneous presence of hierarchy relations among females [17]–[19] and potentially large individual differences in energy requirements. Individual differences in energy requirements are likely to be pronounced because brood-tending females reach an energetic bottleneck due to complete breeding anorexia during incubation [20], and there is substantial variation in both energy reserves at incubation onset and weight loss during incubation [21]. A further advantage is that all brood-tending females have successfully hatched a clutch [22], thus eliminating confounding effects of dominance and reproductive status on leadership propensity. Social foraging is also likely to provide safety benefits due to significant predation pressure on ducklings [23] and adult females [24]. It is most likely because of this predation risk that both vigilance and feeding activities are synchronized among co-tending females, although synchronization may additionally reduce interference between feeding birds such as bumping into each other while diving [25]. First, we determined which variables, reflecting female social status/experience and physiological state, respectively, explained the propensity to initiate foraging bouts, based on a 4-year data set. Second, we assessed the relative importance of the significant social and physiological predictors of the probability of initiating foraging bouts using information theory. Third, for the two years for which such data were available, we determined whether female boldness towards predators [26] had any additional explanatory power over the other predictors of foraging bout initiation.

## Materials and Methods

### Study Area and Species

This study was conducted at Tvärminne (59°50′N, 23°15′E), western Gulf of Finland, in May and June 2004–2005 and 2009–2010. The entire study area is protected through a prohibition on landing on the islands. Suitable foraging sites for broods occur along most shorelines. Brood-tending females provide vigilance and vigorously defend the ducklings against the main duckling predators, great black-backed (*Larus marinus*) and herring gulls (*L. argentatus*). Occasionally broods are also attacked by white-tailed sea eagles (*Haliaeetus albicilla*) posing a threat to both ducklings and tending females. Females may pool their broods and share brood-rearing in coalitions of usually two to five females and their broods, or care for their young solitarily [18]. Brood-rearing coalitions, once stabilized, usually persist for the full brood-rearing period (ca. 40 days; [18]).

### Field Methods

Incubating females were captured by using hand nets. Females were weighed to the nearest 10 g, measured for structural size (length of the radius-ulna in mm), ringed with a standard metal ring, and clutch size was recorded. The length of the radius-ulna is a reliable indicator of female structural size. Thus, based on an extended data set from 1990–2012 (*n* = 2872 observations on 1645 females), projected weight at hatching (see below) was a significant positive function of radius-ulna length, with narrow confidence intervals (*b* = 12.3, 95% CI = 10.7−13.9; linear mixed model with female identity as a random effect and restricted maximum likelihood parameter estimation: *F*_1,1226_ = 222.8, *P*<0.001). Females were also equipped with unique colour rings and temporary wing flags to allow individual identification at sea. The hatch date was estimated using egg flotation [27]. We calculated the number of years since the bird was first ringed and used this as a minimum estimate of years of maternal experience [23], [28]. This is a reasonably accurate age indicator because more than half of the females are trapped annually [29], the fidelity to specific breeding islands is very high [30], and annual trapping effort has been similar since 1996.

We determined the body condition at hatching of all trapped females (*n* = 715, range: 161–220 females annually), provided that they had incubated eggs for >8 days (egg laying may otherwise still be in progress; [21]). As an annual condition index, we used the standardized residuals of a regression of log-transformed projected weight at hatching (response variable) on log-transformed radius-ulna length; indices were derived separately for each year. A female’s weight at hatching was estimated by subtracting an estimate of the weight she would be expected to lose during the remaining incubation time from her measured incubation weight. Each female was weighed once, but because females do not feed during incubation and we captured females at different times in their incubation, we can estimate average weight loss rate during incubation as the slope of the regression of log(body weight) (response variable) on log(incubation time) and projected hatching date [21]. The assumption of continued weight loss after female capture is valid in our study population, and thus our body condition index is reliable [28].

In 2009–2010, we measured the females’ flight initiation distance (FID), the distance (to the nearest 10 cm) between the nest and the approaching person when the female flushed away. FID is a repeatable measure of boldness towards predators [26], despite considerable annual variation in predation risk [30]. FIDs of captured females were measured while re-visiting nests for marking ducklings in conjunction with another study. Nest visits were timed to the estimated hatching of the female’s clutch, based on egg floatation when originally captured (see above); this standardization controlled for the influence of variable incubation time on FIDs [26]. FIDs were always determined by the same person wearing the same clothing, by first ensuring that the bird had noticed the approaching investigator, and then walking towards it following a direct trajectory at a constant low speed, with no obstacles blocking the view between the bird and the observer. Because broods were not always completely hatched at our nest visits, we frequently obtained repeated FID measurements in the same season. Mean annual FIDs were used in the statistical analysis for birds for which repeated measures were available.

Females escort their broods to the water shortly after hatching. Enduring brood-rearing coalitions form on average one week after hatching [31]; being stable for at least two weeks [22]. Enduring coalitions with two (*n = *71), three (*n* = 28) and four females (*n* = 6) were videotaped using digital camcorders (Sony DCR-PC330E and HDR-XR550VE) mounted on spotting scopes, and behaviour was registered using an event recorder program (Etholog 2.25). All focal broods had at least one individually marked, identifiable female, and so we could re-identify broods (*n* = 163 known females (65.2%) out of 250 in 105 broods). We could also distinguish unmarked females by distinct external features [25]. Focal broods were observed for a period of ca. 30 days after nest exodus, with observations lasting up to 1 h per day. The observer was hidden in the landscape and all group members were in the camera’s field of view during filming.

Foraging bouts were defined and timed from the first observation of a female diving until the last bird to feed had surfaced after the last dive; bouts were deemed to have ended if no dives were recorded in the subsequent 5 minutes [25]. We determined the identity of the initiator of each foraging bout (*n* = 380 successful initiations (see below); mean ± standard deviation (SD) per coalition: 3.6±2.5) and the total time spent diving (i.e. actively foraging) by each coalition member during these bouts (*n* = 5647 dives; 23.8±19.3 (mean ± SD) dives per female). To count as successful initiations, the initiator had to be followed by at least one other female (cf. [32]); this was almost invariably (380 out of 399 foraging events, i.e. 95.2%) the case due to high synchronization of diving [25]. Based on a subsample of high-definition video footage from 2010, the median interval between dive initiation and the first female following the initiator was 10.3 s (quartiles 3.1 s, 24.9 s, *n* = 48), which constitutes less than 5% of the average duration of a foraging bout (371 s, see [25]). To quantify individual feeding motivation, we determined the proportion of foraging bouts spent submerged by the focal female for the entire brood-rearing period, hereafter referred to as active foraging time. Using proportional rather than absolute time spent diving allows comparing coalitions which differ with respect to observation time.

Female aggressions and spatial positions were determined for the entire duration of observations (i.e., sampling was not restricted to foraging bouts). All aggressive encounters (*n* = 1292; 12.3±18.2 (mean ± SD) per coalition) and initiator identity were recorded [19]. Female-specific relative aggressiveness was determined as the difference between the observed (*P*_obs_) and expected proportion (*P*_exp_) of aggressive interactions initiated by the focal female, where expected proportions were calculated based on female group size (*P*_exp_ = 1/female group size). For example, if all females in a three-female coalition are equally aggressive, we expect each female to initiate a third (0.33) of all aggressions recorded for the entire coalition. Using a relative measure of aggressiveness removed bias due to brood-specific variation in observation time, the number and age of ducklings, and the frequency of nearby non-group females, all of which influence the incidence of aggression [33]. Determination of female spatial positions has been described elsewhere [19]. To summarize, we determined the position of each female in a coalition relative to the centre of ducklings at 30 s scan-sampling intervals. If females were equidistant to the centre of ducklings, they were assigned a rank that was the mean of the ranks to which each of these females would have been assigned had they not been tied. Scans of females sleeping on land in fixed positions were excluded to minimize serial correlation. Individual mean position ranks and their variances increased with female group size, and hence individual mean ranks were standardized (mean = 0, SD = 1) for each female group size. Positive values of these standardized ranks indicated centrality.

### Data Analysis

The difference between the observed and expected proportions of aggressions was compared after logit transformation to linearize variables. Empirical logits [34] were used for extreme proportions (*P* = 0 or 1), for which the logit is undefined.

We first tested the need for including random effects to account for potential pseudoreplication arising from females belonging to the same brood (*n* = 163 known females in 105 broods) or observed in more than one year (27 out of 163; 16.6%). For this, we constructed a generalized linear mixed model (GLMM) with a binomial error distribution, fitted using Laplace approximation. However, neither brood nor female identity, whether alone or as crossed random effects, explained any of the variation in the probability of initiating foraging. We therefore refrained from including random effects and constructed a generalized linear model (GLM) with a binomial error distribution to assess the effect of our candidate social and physiological predictors on the probability of initiating foraging. This probability was explained by three social factors: female spatial position, relative aggressiveness and breeding experience, and by four physiological factors: female structural size, body condition, active foraging time and reproductive output (clutch size). Female group size has a dual role as it may represent a social factor but also a control variable, since we may expect that the likelihood of initiating foraging should decline with female group size under a null model of random initiation. To clarify the role of female group size in our analyses, we compared the logit difference between the observed proportion of initiations and the expected proportion assuming random initiation for all female group sizes (*P*_exp_ = 1/female group size). The logit difference between these proportions did not deviate from zero for any female group size (one sample *t*-tests; all P>0.20), and thus female group size should be considered a control variable rather than a social factor in our model. Only main effects were included as no significant interactions were found, and non-significant (*P*>0.05) variables were removed. This final model was then subject to residual and collinearity diagnostics to ensure that underlying statistical assumptions were met.

Next, we determined the relative importance of the two predictor categories. This was done by selecting the significant explanatory variables as identified above, assigning them to their respective category and investigating their relative importance by information-theoretic model comparisons [35], in which inference is based on the entire set of plausible models for each of the variable categories (Table 1). Summing the Akaike weights, *ω*, of each model in which a variable occurs, allows a comparison of the relative influence of the explanatory variables. The summed Akaike weights, ∑*ω*, for a given variable will increase with the number of models it is present in. To correct for this, we divided ∑*ω* for each variable by the proportion of the total number of models the variable was part of. Thus, if a variable is present in 4 out of 14 models, the ∑*ω* will be divided by 0.29. This is because if all variables were equally important, their expected ∑*ω* would equal this proportion. If the ∑*ω* of a variable exceeds the expected proportion, it will have a relative *ω* value above 1 and vice versa. These relative *ω* values allow direct comparison of the importance of each variable in explaining the probability of initiating foraging. The relative importance of the two predictor categories was obtained by summing the relative *ω* values for all variables in a given category. Finally, based on a restricted data set (see above), we evaluated whether female boldness (FID) provided additional explanatory power over the explanatory variables included in the final model. All statistical analyses were performed using the software R [36].

**Table 1:** The set of models, constructed from all the significant variables in the final model (see results), used to infer the relative importance of physiological and social predictors of the propensity to initiate a foraging bout.

Model category	Model structure	AIC	ΔAIC	*ω*
P	FS+AC+AF	452.66	0.00	0.40
P	FS+AF	452.77	0.11	0.38
P	AF	455.71	3.06	0.09
P	AC+AF	455.83	3.17	0.08
S	SP+AG	457.62	4.97	0.03
S	AG	459.59	6.94	0.01
S	SP	463.20	10.55	<0.01
P	FS+AC	465.41	12.75	<0.01
P	FS	466.70	14.05	<0.01
P	AC	468.24	15.59	<0.01

The table shows the category of model (physiological, P; social, S), the structure of the model, the AIC value of the model (AIC), the difference in AIC to the best model in this set of models (ΔAIC), and the Akaike weight of the model (*ω*). Abbreviations: active foraging time, AF; female structural size, FS; annual body condition, AC; female spatial position, SP; relative aggressiveness, AG.

### Ethics Statement

Trapping took place predominantly during the end of the incubation period to minimize nest desertion. Potential researcher-induced nest desertion is largely restricted to the early phases of incubation in eiders, whereas the frequency of nest visits does not influence the probability of nest desertion, provided that the first visit is timed to the later phases of incubation [37]. Great care was taken to minimize the time spent on each island during a bout of female capture to further decrease any disturbance. We have also not noticed any adverse effects of the marking techniques involved, such as the use of temporary wing flags on females [38]. Female handling procedures were approved by the Animal Experiment Board/State Provincial Office of Southern Finland, the authority issuing the permit number ESLH-2009-02969/Ym-23 under which we performed this research. Female trapping procedures also complied with the specific regulations of the Tvärminne Zoological Station.

## Results

**Figure 1:**
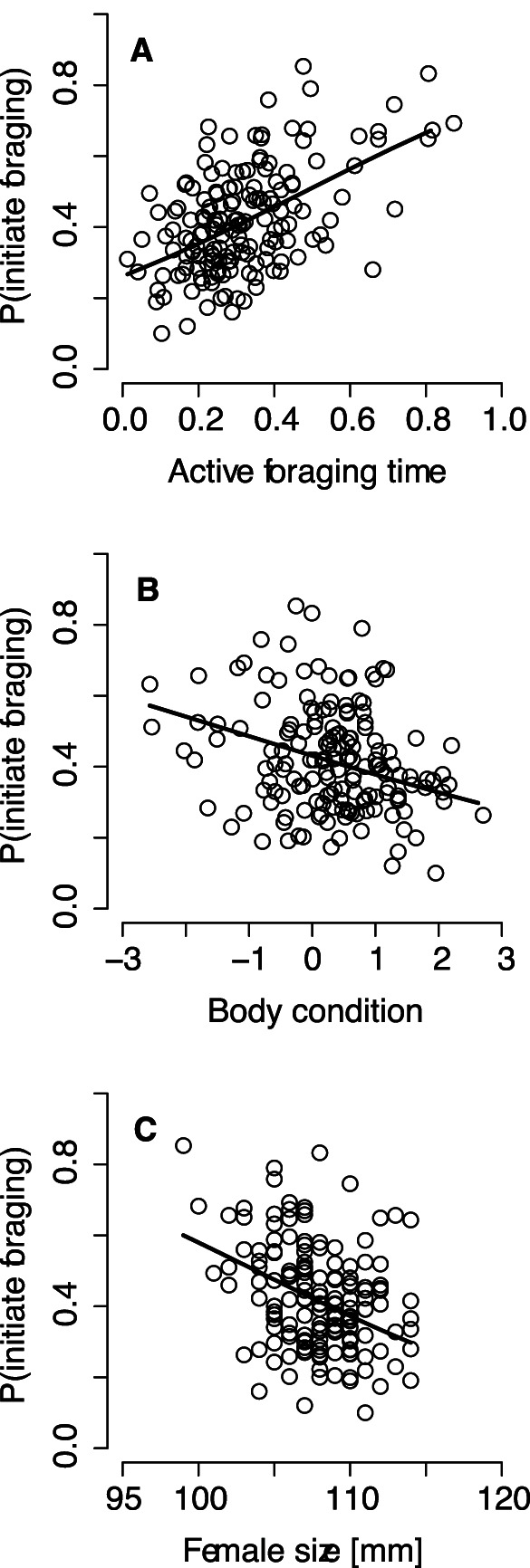
Physiological factors affecting a female’s propensity to initiate foraging in eider brood-rearing coalitions. The propensity to take on leadership in foraging increases with active foraging time (proportion of foraging time spent submerged) (A), decreasing body condition (B) and decreasing structural size (radius-ulna length) (C).

**Figure 2:**
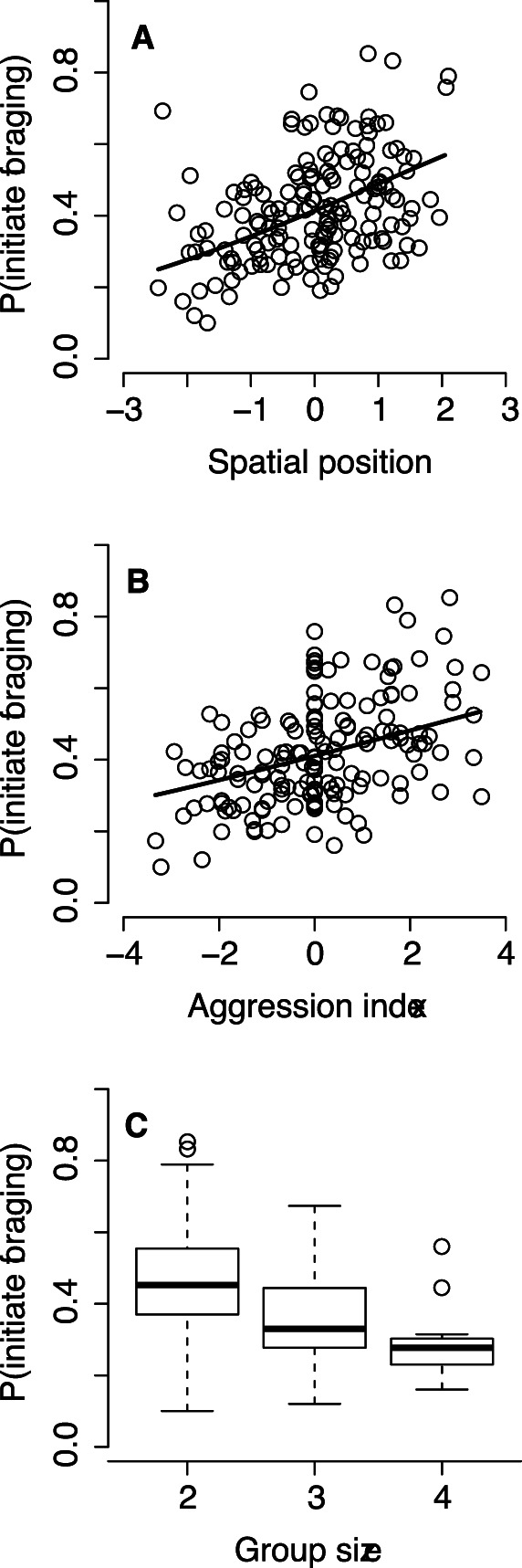
Social factors affecting a female’s propensity to initiate foraging in eider brood-rearing coalitions. The propensity to take on leadership in foraging increases with increasing spatial centrality (A; positive values indicating centrality) and relative aggressiveness (B). The propensity to initiate foraging also decreases with the number of females present in the coalition (C) as expected under random assignment of initiators.

All physiological and social factors except female breeding experience and clutch size affected the propensity to initiate foraging bouts (Table 2). Initiator identity was highly variable, with 73.2% of females (183 out of 250) initiating foraging at least once. With respect to the physiological factors, the likelihood of initiating foraging increased with active foraging time (*b* = 2.16, *z*_156_ = 3.47, *P*<0.0001; Fig. 1A), decreasing body condition (*b* = −0.22, *z*_156_ = −2.17, *P* = 0.03; Fig. 1B) and smaller structural size (*b* = −0.08, *z*_156_ = −2.85, *P* = 0.004; Fig. 1C). As regards the social factors, females initiating foraging bouts had a more central spatial position (*b* = 0.31, *z*_156_ = 2.83, *P* = 0.005; Fig. 2A) and were more aggressive (*b* = 0.14, *z*_156_ = 2.27, *P* = 0.02; Fig. 2B). As expected given random assignment of initiators, the probability of initiating foraging decreased with female group size (*b* = −0.50, *z*_156_ = −3.06, *P* = 0.002; Fig. 2C). No collinearity between explanatory variables was detected (all variance inflation factors <1.16).

**Table 2:** The relative importance of significant physiological and social predictors of the propensity to initiate a foraging bout.

Variable/Category	∑*ω*	Models	Relative *ω*
AF	0.95	4	3.33
FS	0.78	4	2.74
AC	0.48	4	1.70
AG	0.05	2	0.32
SP	0.04	2	0.25
Physiological	0.95	7	1.90
Social	0.05	7	0.10

The table shows the summed *ω* (∑ω), the number of models in which the variable appears (Models), and the relative *ω*, which is calculated by dividing ∑*ω* with the proportion of all models in which the variable appears. Shown are also the ∑*ω* for all models representing physiological and social factors determining leadership, the number of models in the model set representing these factors, and their relative *ω*. Abbreviations: active foraging time, AF; female structural size, FS; annual body condition, AC; female spatial position, SP; relative aggressiveness, AG.

The relative importance of the social and physiological predictors of foraging differed substantially. The importance weights of the physiological factors exceeded those of social factors by an order of magnitude, whether these factors were considered separately or combined (Table 2). Finally, female boldness (FID) did not explain the probability of initiating foraging, either when added to a model containing the six significant predictors of leadership propensity (cf. figs 1 and 2; *b* = −0.000093, *z*_59_ = −0.18, *P* = 0.85), or when included as the sole explanatory variable (*b* = −0.00054, *z*_65_ = −1.15, *P* = 0.25).

## Discussion

Eider brood-rearing coalitions are small, all members can directly communicate with each other, and females establish dominance relationships. In such groups, it has classically been assumed that dominants have priority access to resources, and thus they should initiate foraging activity [6]. Indeed, females that were aggressive and occupied central positions were more likely to initiate foraging. Nevertheless, on direct comparison, the influence of social factors on leadership propensity was overwhelmed by the stronger effect of physiological factors. This predominance may arise because our study system satisfies several key assumptions of the dynamic game models developed by Rands [8], [9]. Thus, most observed brood-rearing coalitions consisted of a pair of females, faced with a simple dichotomous choice of either initiating foraging or following the initiator (cf. [10]). Just as in these theoretical models [8], [9], predation risk appears to be a prime determinant of activity synchronization in female eiders; for example, potentially increased offspring vulnerability to predation results in less overlap in vigilance and diving sequences [25]. Furthermore, individual differences in energy reserves and requirements are likely to be pronounced in post-incubating energetically challenged females, and hence they should be differently motivated to forage (cf. [15]). By initiating foraging, animals may manipulate their daily foraging efficiency [39]. Accordingly, active foraging time was the single most important predictor of foraging bout initiation (Table 2, Fig. 1A). It should be noted here that a greater active foraging time is not an inevitable by-product of foraging initiation. Thus, initiators only gain a short head start compared to their coalition partners (see Materials and Methods), and therefore followers should, if need be, easily be able to catch up with the initiator regarding their diving time, e.g. by ending their foraging slightly later or by increasing their dive frequency. Because this usually does not occur, this finding adds to the slowly accumulating evidence that ‘hungry’ individuals may act as pace-makers in social foragers [9]. In contrast, our finding that structurally smaller females were more likely to act as initiators seems, at first sight, at odds with the notion that larger individuals may have greater energetic demands [16]. However, physically smaller individuals are constrained in their capacity to store energy and have higher-mass specific metabolic rates, which may force them to forage more frequently (e.g. [40]).

The models proposed by Rands [8], [9] predict that hungry individuals rarely catch up other group members who have larger energy reserves, and thus individuals tend to become locked into leader and follower roles persisting for some time. This prediction has to our knowledge never been directly tested. In this respect, it is pertinent that females in poor body condition at hatching (i.e., having smaller energy reserves) were more likely to initiate foraging (Fig. 1B), even though their foraging behaviour was observed several days, sometimes even weeks, after the hatching of their broods. The highly synchronous foraging behaviour of female eiders both during [25] and outside [41] the brood-rearing period may therefore contribute to the maintenance of individual differences in body condition even over longer time scales than we have studied here. Thus, individual body condition in female eiders shows high annual repeatability (female identity explains over 40% of the total variation; [29]), and body condition at the first documented breeding attempt is, in turn, positively correlated with future survival [42].

Our study stands in apparent contrast to the evidence suggesting that boldness may predispose animals to the leadership role [2], [43]–[45]. One possible reason for this discrepancy may be that most studies that found effects of boldness on leadership propensity defined boldness using responses to novel objects or environments rather than to threatening stimuli, as we have done here. As demonstrated by Carter et al. [46], these two types of boldness measures may not even be correlated with each other and instead reflect different personality dimensions. Alternatively, individual differences in satiation level among eider females may be strong enough to mask any effects of personality differences on leadership propensity. For example, Nakayama et al. [12] showed that experimental feeding of bolder sticklebacks (*Gasterosteus aculeatus*) actually led to a role reversal, with the shyer fish emerging as leaders. There is also an inherent danger in predicting the outcome of behavioural interactions in groups on the basis of boldness measures determined when the animal is isolated from others. For example, the possible role of individual boldness in affecting the tendency to initiate group activities may be shaped by the mix of personality types in the group [45].

Female breeding experience did not affect the propensity to initiate foraging. Although individuals may be more likely to become leaders owing to the amount of information they possess [47], information asymmetries should only be important if the difference in information is large [48]. This is unlikely here, since the invertebrate prey of eider broods, gammarid shrimps *Gammarus* spp. and blue mussels *Mytilus edulis*, is widely distributed and abundant [49]. However, the non-significant effect of female age on leadership propensity is nonetheless interesting because we have recently demonstrated that the relative frequency of relatives encountered by a female increases with advancing age [50]. In socially-structured societies, the ability of leaders to elicit follower behaviour may depend on the relative number of kin relations, with individuals preferring to follow related individuals [51]. Acknowledging that a female’s age is at best an imperfect proxy for the size of her matriline in a brood-rearing coalition, further research specifically aimed at investigating the distribution of leadership in relation to group kin structure is warranted.

Recent theoretical work suggests that in contrast to strategies such as despotic leadership, ‘leading according to need’ is an evolutionary viable strategy whatever the group heterogeneity [52], and thus dominance rank may be an uninformative criterion for predicting leadership even when strict dominance hierarchies occur [5]. Our results lend empirical support to these ideas, by showing that when weighed against each other, physiological need may override the influence of pre-existing individual properties such as social status.
